# Developing a Realistic and Cost-Effective Training Model (MaiSurge) for Laparoscopic Hysterectomies to Train and Assess Surgical Skill: Prospective Nonrandomized Controlled Trial

**DOI:** 10.2196/66369

**Published:** 2026-02-12

**Authors:** Anna Maria Brechter, Roxana Schwab, Christoph Dold, Christine Skala, Maria Schröder, Lina Schiestl, Katharina Gillen, Walburgis Brenner, Annette Hasenburg, Mona Wanda Schmidt

**Affiliations:** 1 Department of Obstetrics and Gynecology, University Medical Center of the Johannes Gutenberg University Mainz, Mainz, 55131, Germany; 2 Department of Gynecology and Gynecologic Oncology, Diakonie Hospital Jung-Stilling, Siegen, Germany

**Keywords:** surgical education, laparoscopic hysterectomy, training model, surgical skill, assessment, surgery, hysterectomy, cost-effective, training, laparoscopic surgery, salpingectomy, adenectomy, electrosurgery, gynecologists, gynecology, patient safety, controlled trial

## Abstract

**Background:**

Laparoscopic surgery has a flatter learning curve compared to traditional open surgery. Therefore, structured programs and realistic training models are imperative to ensure patients’ safety. However, commercially available models are often too expensive or technically unrealistic for continuous surgical training.

**Objective:**

The aim of this trial was to develop a cost-efficient and highly realistic uterus model to perform a total laparoscopic hysterectomy (TLH) and evaluate its applicability.

**Methods:**

A training model (MaiSurge) for a TLH with salpingectomy or adenectomy was developed using a 3D printer and different cast materials. Polyvinyl alcohol was used to allow for the use of electrosurgery. To gather the first validity evidence, novice and expert gynecologists performed a TLH on the model. Operative time as well as surgical performance scores (Hysterectomy–Objective Structured Assessment of Technical Skills) were compared between both groups.

**Results:**

A total of 12 participants in the novice group and 18 participants in the expert group completed the simulation. The experts obtained significantly better modified Hysterectomy–Objective Structured Assessment of Technical Skills scores (mean 74.0, SD 12.9 vs mean 60.3, SD 14.9; *P*=.049) and performed significantly faster (median 69.5, IQR 49.5-74.3 minutes vs median 37.5, IQR 30.5-38.8 minutes; *P*<.001). An excellent interrater reliability was observed (intraclass correlation coefficient=0.91). Approximately 92% (11/12) of novices felt that they had improved their surgical performance after training on the MaiSurge uterus model. Overall, all participants agreed that the new MaiSurge uterus model should be integrated into training curricula to improve the performance of residents on TLHs.

**Conclusions:**

A new highly realistic and cost-effective training model (MaiSurge) to perform a TLH was developed. The model distinguishes between good and poor laparoscopic performances and, thus, can be used in training as well as assessment of surgical skills. The possibility of simulating even complex laparoscopic procedures in a realistic environment may be an opportunity to train a future generation of gynecologists without compromising patient safety or exhausting the limited availability of operating room time.

## Introduction

A hysterectomy is a common procedure in gynecology in which the uterus and, in the case of an additional salpingectomy, the fallopian tubes are removed [1]. In 2016, the prevalence of hysterectomies was approximately 21% of approximately 600,000 hysterectomies performed overall each year in the United States [2,3]. The procedure can be performed laparoscopically, vaginally, or per open surgery. While intraoperative complications seem to be comparable between those approaches [4,5], the loss of depth perception, the indirect view on the operating field, and the fulcrum effect including the inversion and scaling of movements in laparoscopic surgery are associated with prolonged learning curves [6]. Training residents in surgical fields is challenging while simultaneously trying to maintain patient safety and keep operating costs low in light of the increasing financial pressure on hospitals. Recent studies have shown that the complication rate and outcomes of total laparoscopic hysterectomies (TLHs) performed by residents are not worse compared to those of TLHs performed by senior physicians, but that the operating time is significantly longer (16%-32% increase in operative time) [7,8], which drastically increases costs for the hospitals and anesthesia times for patients. Currently, gynecology residents in Germany are still learning how to perform laparoscopic surgeries directly in the operating theater on the patient. Simulation training is rarely included within structured residency training programs, and there are no criteria that a resident must meet before performing the first procedure on a patient. These prolonged operative times can result in economic damages to the hospital and disadvantages for the patient due to the longer anesthesia time. Therefore, shortening the learning curve through skill laboratory training can positively impact not only patients but also the training of residents and financial aspects for hospitals.

Apart from numerous basic laparoscopic skill training models, which have been shown to improve surgical performance [9], there is a lack of effective, feasible, and cost-efficient training models for more complex skills. The critical steps of a surgical procedure, as well as full surgical procedures themselves, need to be trained [10]. Currently, there are only a few inanimate models of the uterus on the market, but these are expensive and mostly nonconductive and, thus, unsuitable to use with electrocautery. Animal cadavers, on the other hand, require specialized facilities, and the anatomical differences limit the transferability to humans. To simulate hysterectomies for continuous practice, some researchers have built very simple models from materials such as balloons and plastic tubes [11]. However, due to the materials used and their simplicity, these models are not realistic enough to simulate all surgical steps. Many surgical specialties are faced with similar challenges. To overcome them, some have used 3D printing technologies to create training models, for example, in pancreatic surgery [12]. However, no comparable models are available for laparoscopic hysterectomies.

Thus, the aim of this study was to develop a feasible uterus model to practice laparoscopic hysterectomies outside of the operating room and that can reflect the current surgical skill of a gynecological resident. A main focus was to create a model that addressed the aforementioned shortcomings of the available models by being realistic and cost-effective and allowing for electrosurgery to be used. This study focused on the question of whether the newly developed uterus model can differentiate between good and bad surgical performances and whether it is perceived to adequately reflect a real TLH on a patient. To evaluate this, we conducted a prospective, nonrandomized controlled trial. We hypothesized that the newly created uterus model would convince gynecological experts of its realism and be able to differentiate between levels of surgical expertise based on procedure time and surgical performance scores. If proven true, the newly developed uterus model could be used for continuous surgical education of gynecological residents worldwide, allowing inexperienced surgeons to have their first experience with a TLH and also allowing for their skill level to be assessed and the learning curve to be observed. Especially in areas where minimally invasive surgery is not performed every day due to restricted availability, cost-effective training models such as this uterus model could impact patient safety immensely.

## Methods

### Development of the MaiSurge Uterus Model

Different uterus models were developed using 3D-printed molds, which were then cast with silicone and biotissue material. The uterus model was designed to meet the following criteria: similar texture to human tissue, applicability for training, similar optics to human situs, and realistic surgical procedure performance. Different materials and molds were tested throughout the development stage and adapted based on expert feedback.

**Figure 1.**
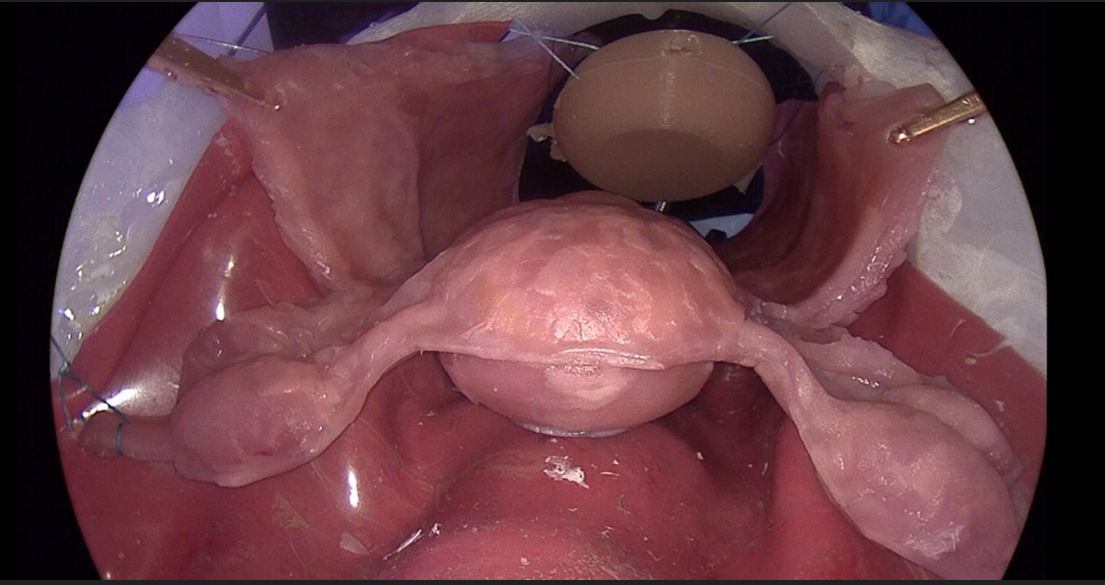
MaiSurge model—newly developed uterus model made from polyvinyl alcohol.

The molds were created using the 3D software programs Blender (Blender Foundation) and Autodesk Fusion 360 (Autodesk). For dimension and shaping, pictures and videos, as well as data from textbooks, were used. The 3D datasets of the molds were then printed using the Original Prusa i3 MK3S+ (Prusa Research) 3D printer and injected with silicone or polyvinyl alcohol (PVA). Two different degrees of hardness were used to simulate the layers of the tissue in the silicone model. Models made of PVA underwent several frosting and defrosting cycles until they reached the desired consistency. The uterus model consists of the corpus and the cervix of the uterus with the vagina, as well as 2 tubes with fimbria; 2 ovaries with the utero-ovarian ligaments, the round ligaments, and the broad ligaments; and a layer of peritoneum and the uterine arteries. The corpus and the cervix of the uterus and the vagina are hollow inside so that an adapter can be inserted and the hysterectomy can be performed on it. The newly developed MaiSurge (PVA) uterus model (Figure 1) was used for further evaluation in the trial as it offers the opportunity to work on it using electrocautery during the surgical procedure. The model was fabricated in 3 steps: one for the inner layer, one for the outer layer and the adnexa, and one to add the ventral part of the visceral peritoneum.

### Setting and Participants

This study was conducted in the training center for minimally invasive surgery of the Department of Obstetrics and Gynecology at the University Medical Center of Johannes Gutenberg University Mainz. Residents and attending gynecologists of the Department of Obstetrics and Gynecology at the University Medical Center of Johannes Gutenberg University Mainz were recruited for the trial and categorized according to their prior experience with TLH in the last 5 years (experts: ≥10; novices: <10).

### Ethical Considerations

This study was registered at the German Clinical Trials Register (DRKS00031825), and ethics approval was granted by the ethics committee of the Rheinland-Pfalz medical association (2022-16451_1). Written informed consent was obtained from all participants before the start of the trial. Participants received no compensation for participating in this trial All data was recorded pseudonymised, with only the participant being able to identify their pseudonym retrospectivley.

### Study Design

This was a prospective, nonrandomized, rater-blinded controlled trial. A flowchart of the study design can be found in Multimedia Appendix 1. All participants performed 3 Z-sutures on a vaginal cuff model (with or without prior video explanation) before being shown a video of the TLH procedure to be performed. Subsequently, all participants performed a TLH on the newly developed uterus model with the camera and instrument assistance of a trained tutor. The tutor only acted on direct instruction of the trainee. All performances were video recorded and evaluated by 2 blinded raters.

### Surgical Procedure

As the first step of the surgery, the trocars were inserted in the holes of the training box. Starting with the coagulation of the utero-ovarian ligaments, the salpingectomy was performed. Coagulation was achieved through a bipolar grasper and cutting with simple nonelectrical scissors. The round ligament was coagulated completely and then cut. The ventral and dorsal layers of the broad ligament were separated to expose the uterine artery, which appeared after approximately 2 to 3 cm. The uterine artery was then coagulated and cut. After the identification of the vesico-uterine peritoneum, a horizontal incision was used to create a bladder flap and dissect the bladder from the lower uterine segment and cervix. To detach the uterus from the vagina, a colpotomy was performed along the vaginal adapter using a monopolar hook. Because, in this simulation, the extraction through the vagina was not possible, the uterus was removed from the top. The vaginal cuff was closed using Z-sutures. No specific number of sutures on the vaginal cuff was required; rather, the participants were asked to place as many as needed to ensure full closure of the vaginal cuff.

### Outcomes

The primary outcome was the quality of the surgical performance, assessed using a modified Hysterectomy–Objective Structured Assessment of Technical Skills (H-OSATS) [13] score, which was developed specifically for the laparoscopic hysterectomy. This score assesses the complete performance of the operation, including positioning of the patient; access to the abdominal cavity; inspection of the organs; insertion of the trocars; and all steps of the operation, such as cutting of the ligaments, separation of the uterus, suturing, and the entire withdrawal of the uterus. In this case, 1 to 5 points (Likert scale) can be awarded depending on how well the surgical step was performed. To fit the simulated procedure, the original H-OSATS score [13] was modified by removing several items, such as the identification of some anatomical structures in the pelvis, which were not included in the model. The modified version can be found in Multimedia Appendix 1. A total of 21 items were included, leading to a maximum of 105 points. Furthermore, a commonly used Objective Structured Assessment of Technical Skills (OSATS) score (global rating checklist, 7 items on a 5-point Likert scale) by Martin et al [14] was used to assess procedure-independent laparoscopic skills. The H-OSATS score was assessed by 2 specifically trained, blinded raters based on video recordings of the procedure. The operating time was evaluated as a secondary outcome. Furthermore, a 5-point Likert scale was used to evaluate the MaiSurge (PVA) model, the silicone model, and a commercially available uterus model with regard to texture, simulation quality, and realism. The baseline suturing and knot-tying attempts were scored based on the time taken to perform the sutures, as well as using the suturing and knot-tying–specific OSATS score by Chang et al [15]. Among other things, the score assesses how the needle is clamped, how it is guided through the tissue, how the knots are made, and whether the needle remains in the camera’s field of vision. In addition, the handling of the instruments and the tissue, as well as the independent performance of the procedure, is assessed.

### Statistical Analysis

Descriptive analysis and graphical illustrations (created using SPSS version 27; IBM Corp) are reported where appropriate. Categorical data are presented in absolute and relative frequencies, and continuous data are presented as medians and IQRs or medians and SDs where appropriate. The evaluation of the uterus model was performed using a 5-point Likert scale, which is reported as median and IQR or as a percentage of agreement (4 or 5 points on the Likert scale). The Shapiro-Wilk test was used to test for normality. Primary and secondary outcomes were compared between experts and novices using the Mann-Whitney *U* test (nonparametric) or an independent 2-tailed *t* test (parametric). Interrater reliability was assessed using the intraclass correlation coefficient (ICC) for the modified H-OSATS score and the OSATS score. ICCs of less than 0.5 were regarded as poor, ICCs between 0.5 and 0.75 were regarded as moderate, ICCs between 0.75 and 0.9 were regarded as good, and ICCs of >0.9 were regarded as excellent [16]. A 2-sided *P* value of less than .05 was considered statistically significant. Statistical analysis was performed using Stata/BE (version 17.5.0; StataCorp) and SPSS (version 27).

## Results

### Study Population

A detailed summary of the baseline characteristics is shown in Table 1.

In addition to the baseline data, the prequestionnaire asked to what extent the participants were confident that they could perform a TLH without major errors. This question was answered with a median of 1 (IQR 1-1) (“disagree”) in the novice group and a median of 5 (IQR 4-5) (“agree”) in the expert group using a 5-point Likert scale.

**Table 1.** Baseline characteristics.

	Novices (n=12)	Experts (n=8)
Age (y), median (IQR)	30 (28.4-33.8)	42 (33.2-49.8)
Gender, n (%)		
Woman	10 (83.3)	5 (62.5)
Man	2 (16.7)	3 (37.5)
Educational level, n (%)		
Resident	8 (66.7)	0 (0.0)
Specialist	3 (25.0)	2 (25.0)
Attending	1 (8.3)	6 (75.0)
Work experience (y), median (IQR)	3.75 (0.5-7.3)	15 (6.25-23.3)
Prior training on surgical simulator, n (%)	7 (58.3)	6 (75.0)
Prior training course in MIS^a^, n (%)	3 (25.0)	6 (75.0)
Number of laparoscopic procedures seen, median (IQR)	50 (28.75-250)	590 (300-2500)
Number of laparoscopic procedures assisted, median (IQR)	30 (5.25-87.5)	300 (200-1700)
Number of laparoscopic procedures performed, median (IQR)	7.5 (0-50)	225 (125-1675)
Number of TLHs^b^ performed overall, median (IQR)	0 (0-0.75)	75 (11.5-150)
Number of TLHs performed in the last 5 years, median (IQR)	0 (0-0.75)	40 (10.5-100)

a MIS: minimally invasive surgery.

b TLH: total laparoscopic hysterectomy.

### Suturing and Knot Tying on the Vaginal Cuff Model

The 3 suture and knot attempts on the vaginal tube model were assessed using the OSATS score [15]. The experts performed significantly faster (*P*<.001) and scored significantly better (*P*<.001; for details, see Multimedia Appendix 1).

### Primary Outcome

**Figure 2.**
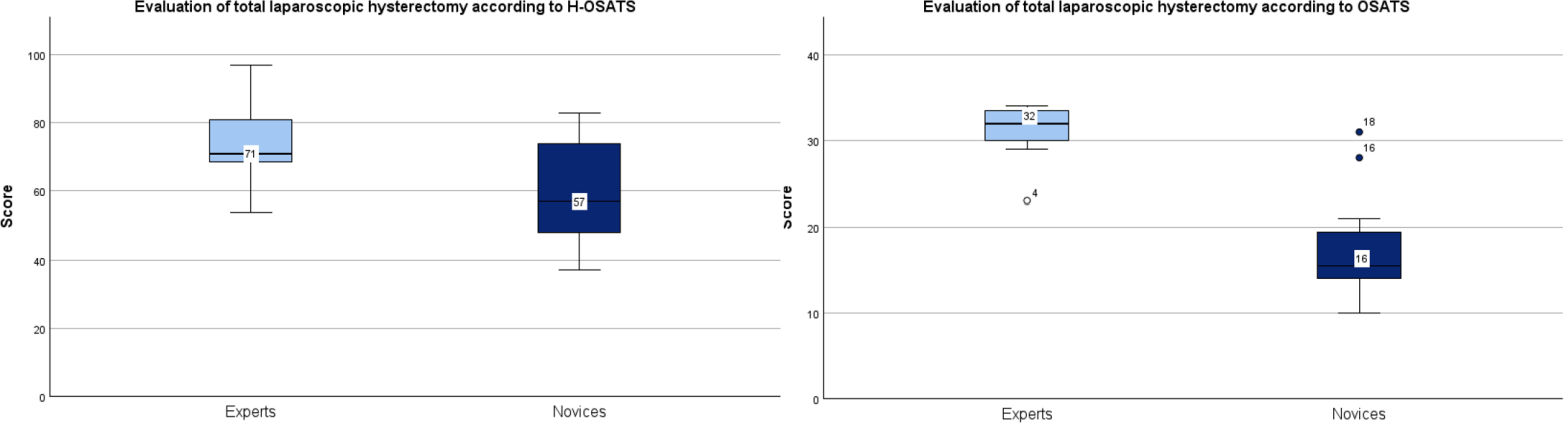
Comparison of Hysterectomy–Objective Structured Assessment of Technical Skills (H-OSATS) score and general Objective Structured Assessment of Technical Skills (OSATS) score between experts and novices.

The expert group obtained significantly higher procedure-specific modified H-OSATS scores compared to the novices (mean 74.0, SD 12.9 vs mean 60.3, SD 14.9; *P*=.049). Similar results were observed for the general OSATS score (*P*<.001), with a median of 71 (IQR 68.5-81) versus 57 (IQR 48-74; Figure 2). An excellent interrater reliability was observed for the H-OSATS score, with an ICC of 0.91 (*P*<.001), and a good interrater reliability was observed for the OSATS score, with an ICC of 0.81 (*P*<.001).

### Operating Time

**Figure 3.**
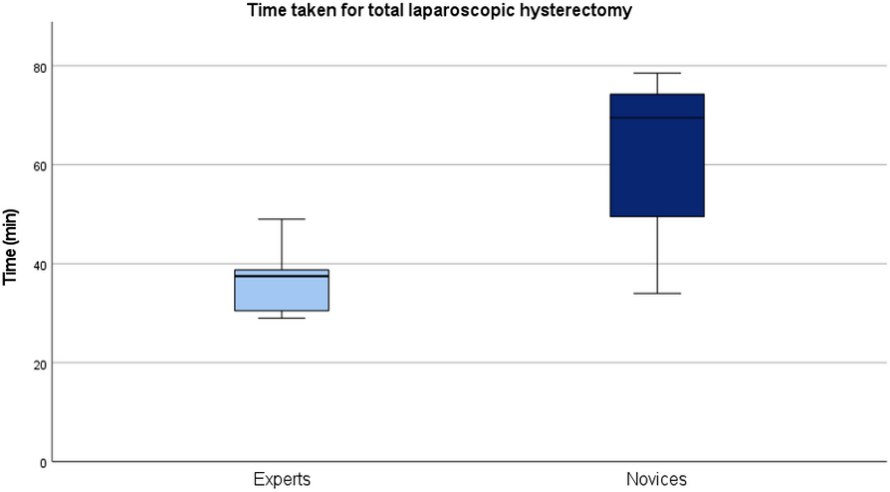
Comparison of operating time for a total laparoscopic hysterectomy between experts and novices on the MaiSurge model.

Significantly prolonged operating times were recorded in the novice group compared to the expert group, with an 85.3% increase in operating time (novices: median 69.5, IQR 49.5-74.3 minutes; experts: median 37.5, IQR 30.5-38.8 minutes; *P*<.001; Figure 3).

### Subjective Evaluation of the Uterus Model

**Figure 4.**
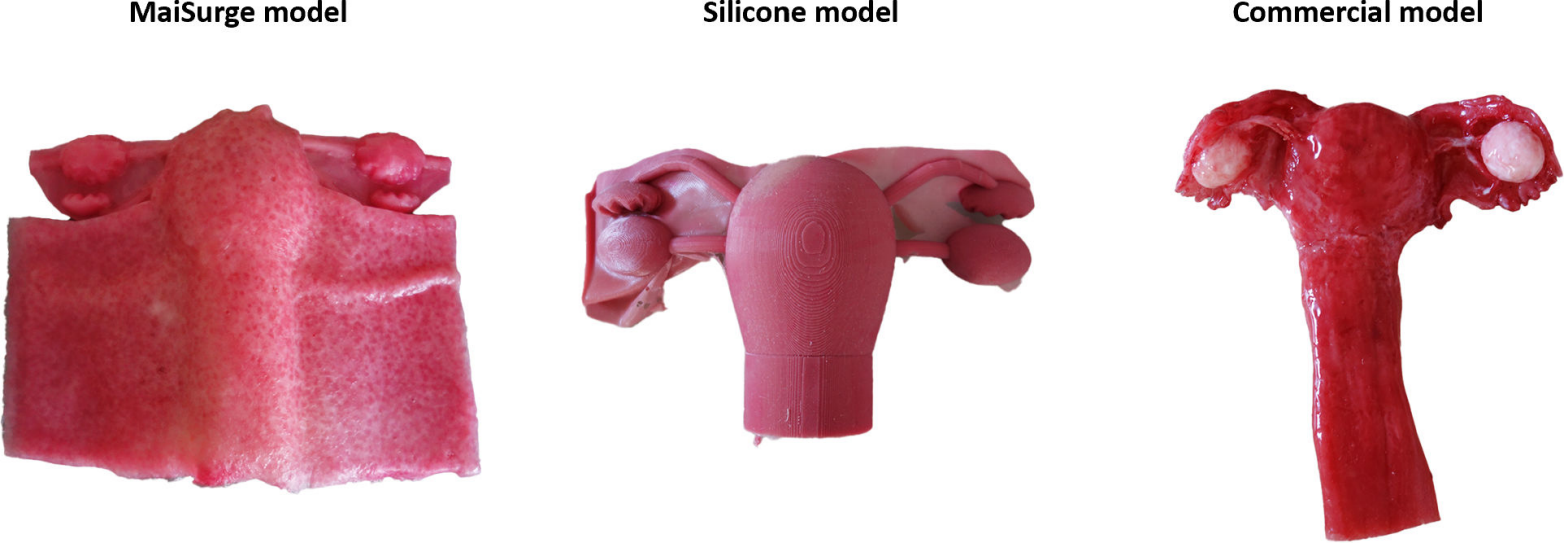
Evaluated training models.

**Figure 5.**
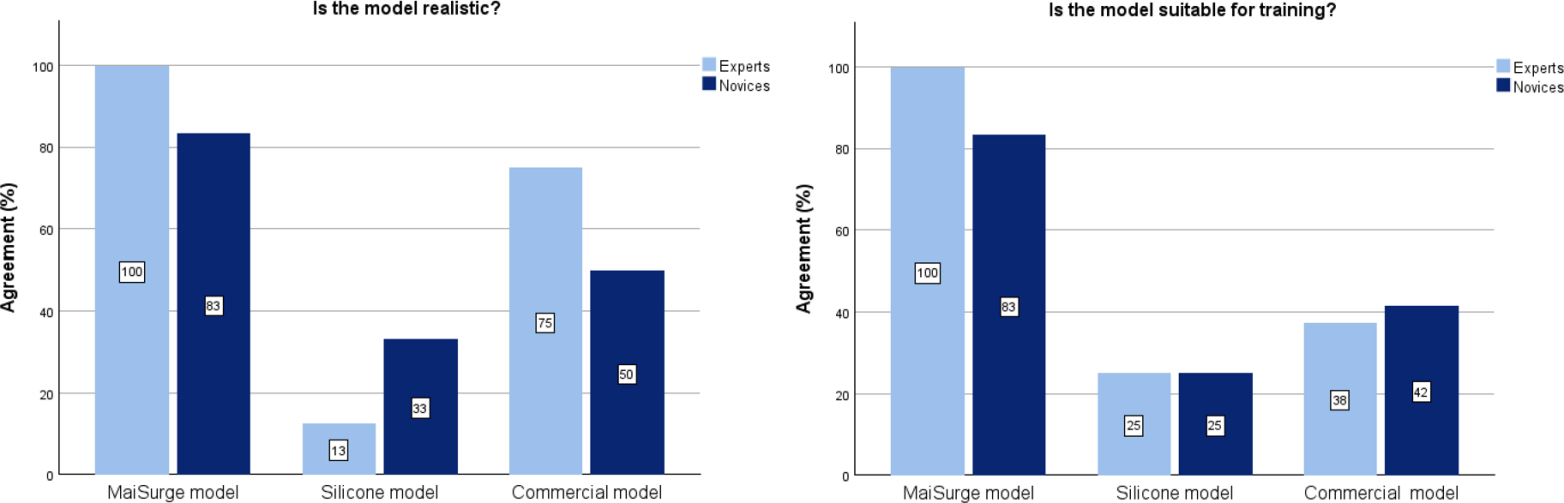
Comparison of different training models in terms of realism and suitability for training.

The newly developed MaiSurge uterus model made of PVA was compared to the same model made from silicone, as well as a commercial uterus model from MedicFX (uterus and ovaries; Figure 4). Overall, the newly developed model was rated better or equally well compared to the silicone model and the commercial uterus model with regard to realism, color, consistency, size, thickness, tensile strength, proportions, and suitability for training (Figure 5).

In terms of consistency, experts in particular identified areas for potential improvement of the MaiSurge model to increase the realism of the texture as it was often perceived to have less tensile strength compared to the real uterus tissue. The experts rated the model with a median of 3.5 (IQR 3.0-4.0) points on the Likert scale. In this regard, the commercial model scored slightly better, with a median of 4.0 (IQR 3.0-4.0) points. The novices gave the MaiSurge model a median score of 3.5 (IQR 3.0-4.0) points, with the other 2 models scoring lower.

Experts awarded the MaiSurge model a median score of 4.0 (IQR 2.25-4.0) points for tear resistance and, therefore, rated the model better than the other 2. Novices awarded the MaiSurge model a median score of 2.5 (IQR 1.25-4.0) points, which was considerably lower than the score from the experts.

**Figure 6.**
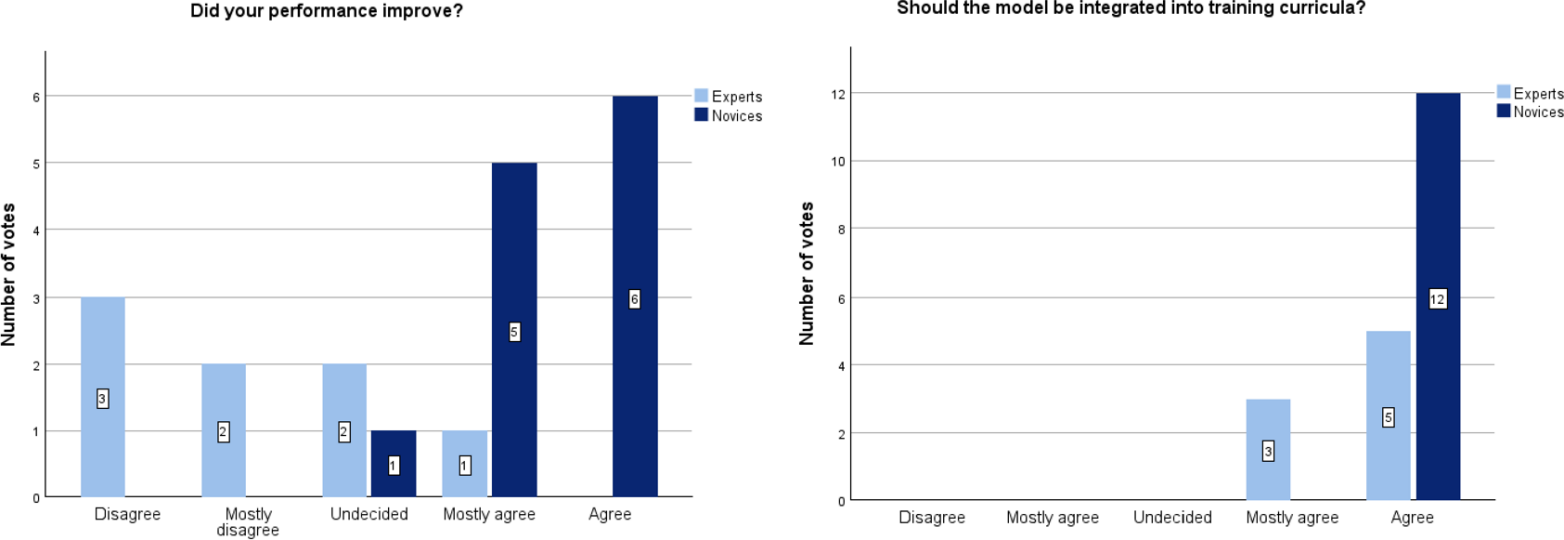
Evaluation of subjective perception of skill improvement and use of the MaiSurge model for future training.

While only 12.5% (1/8) of the experts reported improvement in their performance, approximately 92% (11/12) of the novices acknowledged that they had improved their surgical performance after training on the MaiSurge model. Overall, all participants agreed that the new MaiSurge uterus model should be integrated into training curricula to improve the performance of residents on TLHs (Figure 6).

## Discussion

In this study, the MaiSurge uterus model was developed and evaluated to realistically simulate a TLH.

### Principal Findings

On the newly developed MaiSurge model, experts not only performed the TLH significantly faster than residents and fellows (approximately 85% longer operative times for the latter) but also achieved significantly higher performance scores as measured using the modified H-OSATS (approximately 23% higher scores) and general OSATS (approximately 24% higher scores) scales. Furthermore, the MaiSurge model can be used with conventional mechanical surgery as well as with electrosurgery and, thus, was rated as highly realistic by experienced gynecological surgeons and gynecological residents, especially when compared to widely available silicone models. All participants who completed the simulation TLH on the MaiSurge model agreed that the model was realistic in terms of color, size, thickness, proportions, and suitability for training. For inexperienced surgeons, the brittleness of the material posed a difficulty when suturing the vaginal stump as the model tended to tear out during “rough” movements. In addition, the thickness of the stump was an obstacle as it was difficult to push the needle through the material. However, despite the obstacles, the expert group was able to perform this surgical step without major problems. Furthermore, participants unanimously found the model to be a valuable tool to train residents, demonstrating its value and need in today’s education of a future generation of gynecological surgeons.

### Implications of the Findings

The number of gynecology inpatient procedures in Germany has declined in recent years, and the trend is likely to continue [17]. In 2023, the surgical catalog of outpatient procedures in Germany was adjusted to include more procedures that should be performed as outpatient procedures [18]. As a result, fewer procedures suitable for acquiring basic surgical skills will be performed in hospitals. Thus, especially in university hospitals, the number of training procedures per resident will decrease as the remaining surgical procedures are often too complex to be considered training procedures. Combined with the decreasing working hours due to improved labor laws, it will be difficult to fully guarantee adequate surgical training [17,18]. With the MaiSurge model, a realistic and affordable training tool was created to facilitate the surgical education of gynecological residents and fellows outside of the operating room. Regarding training model validity, evidence is needed before its implementation in training curricula. In this trial, gynecological experts outperformed novices with regard to time and performance scores, indicating that surgical skills when performing a TLH are adequately reflected through the use of the MaiSurge model. This provides attending physicians and program directors with the possibility to assess the current skill level of a gynecological resident. Furthermore, due to its ability to reflect surgical skill and its realism, the MaiSurge model could potentially be used as a test before residents are allowed to operate on a human patient. However, to achieve this, a benchmark with regard to time and performance scores needs to be set, which will require further research to evaluate a meaningful cutoff. We strongly believe that the MaiSurge model will be a necessary and feasible training tool to address the changes in surgical education ahead of us.

As expected, novices and experts found the model to be useful for the training of a TLH outside of the operating room. Novices reported a noticeable improvement in their performance after using the MaiSurge model. In contrast, experts did not report the same improvement as expected. However, this highlights that the MaiSurge model should be used at the beginning of a trainee’s surgical learning curve rather than when a certain competency is reached so that they can benefit the most from the simulation. Since 2014, dry laboratory training is required by the European Union of Medical Specialists in every hospital offering laparoscopic procedures [19]. However, this is not yet a reality, which could be due to the required financial investments and lack of advanced training models. Thus, the MaiSurge uterus model is a good step toward meaningful and feasible dry laboratory training. As demonstrated by Klapdor et al [20], a structured skill laboratory training curriculum for gynecological residents showed a 16% decrease in operative time for a TLH after only 1 year. However, in the latter trial, a general laparoscopic skill training curriculum was used rather than a realistic uterus model, which leaves us imagining the potential impact that a realistic and procedure-specific training (eg, using the MaiSurge model) can have. This assumption is supported by the results of the study by Yang et al [10], who showed that a specific model for a pancreatoduodenectomy and training is better than just basic laparoscopic skill training.

### Comparison to the Literature and Other Training Models

Basic laparoscopic skills can be taught through a variety of available models and trainers, which can be purchased for approximately €4000 (US $4697.44) (eg, the Lübeck Toolbox) [21], or by building one’s own [22]. However, for more complex skills, advanced training models are needed. While some models for advanced skills, such as the closure of a vaginal cuff, can be cost-efficiently created from widely available building materials [23-25], they often lack realism and, thus, limit the extent of transferable skills to acquire. Purchasable options are often more realistic. However, they come at a price per vaginal cuff model that is often not suitable for large-scale training, such as those by LifeLike BioTissue (US $50) [26,27], Faux Medical (US $45) [28], or Medarchitect (US $40) [29]. Although it makes sense to train the surgical step of closing the vaginal cuff separately, a training model is needed that can cover as many surgical steps as possible to increase cost-efficiency. Only a few models for TLHs are available for purchase, such as a model from the company Oniko [30]. In addition to the high cost, this model is made of silicone and cannot be used with electrosurgery, which greatly impacts the realism of training and, thus, can potentially impact the skill transfer to the real operating room. To our knowledge, there is only 1 purchasable model from Limbs & Things that allows for electrosurgery during a TLH, which has an even higher cost of US $640 per uterus [31] and, thus, is not feasible for continuous training as only a few residency programs can provide each resident with multiple models at this cost.

With the MaiSurge model, some of these disadvantages of simulation models have been addressed. The MaiSurge model allows for the use of electrocautery, provides haptic resistance, and is relatively inexpensive to produce. The MaiSurge model developed in this study costs approximately €30 (US $35.23) (50 g of PVA per model) for the material plus 5 hours of work time (2 hours for boiling, 1 hour for casting the first layer, 1 hour for casting the second layer, and 1 hour for the peritoneum and vessels (boiling and casting); possibly less if mass produced). Once mass production is established, those costs will go down even further. Therefore, this model could be used in training courses, as well as for consistent training in residency programs.

### Strengths and Limitations

The strengths of this study include the prospective, controlled trial design. Furthermore, 2 trained and blinded raters evaluated the primary outcome via video recordings, thus reducing the risk of assessment bias. A high interrater reliability was found, demonstrating the reproducibility of our results. With 75% (18/24) of the eligible physicians at the facility participating in the trial, including residents from every stage of training [32], we believe that the data presented in this study are representative of a university hospital in Germany and clearly reflect the potential use of the MaiSurge model to differentiate between experienced and inexperienced surgeons. However, it should be noted that the sample size of this trial was rather small, posing a risk of bias, but overall, the sample size is comparable to those of other studies developing new training models [23,33,34]. A follow-up multicenter trial is planned to assess transferability to the operating room. While the realism of the simulation of a TLH on the new MaiSurge model was rated highly, there are aspects in which the realism and, thus, the training experience could be further increased. This includes the simulation of blood flow in the model, as well as the possibility of removing the uterus vaginally (eg, if necessary with morcellation). Ultimately, the performance of the expert group could have been compromised by the few less realistic features of the model (eg, the traction on the tissue, which might have taken time to adjust to). While not explicitly assessed in this trial, a transfer of skills to the operating room can be expected based on current evidence of the transferability of surgical skills acquired in a skill laboratory and the high rating on realism and suitability from experienced gynecologists in this trial. Future randomized controlled trials could not only focus on the effect of training using the MaiSurge uterus model on intraoperative performances but also compare the training effects of the MaiSurge uterus model with those of commercially available and more expensive models such as the model by Limbs & Things [31]. While we were able to demonstrate the value of the MaiSurge uterus model in this trial, no objective comparison to similar and more expensive uterus models was drawn due to financial limitations.

One major advantage of the MaiSurge uterus model is the possibility to perform salpingectomies, adnexectomies, and TLHs on the same model, allowing inexperienced and experienced trainees to share a model to address their respective stages of training. Currently, the model is being developed further to include simulated bleedings, as well as uterine fibroids for laparoscopic and hysteroscopic resection. This will enhance the usefulness of the MaiSurge uterus model for more advanced training (eg, during fellowships or early stages of being an attending physician). Potentially individualized uterus models can be created based on real magnetic resonance imaging scans to simulate extremely difficult procedures and train a specific case before performing the same procedure on the actual patient. While this trial offers the validity evidence needed to use the MaiSurge model to assess a trainee’s skill level, other questions remain unanswered. Future studies will need to focus on the learning curve of trainees on the MaiSurge model, the evaluation of other surgical procedures (eg, myomectomy or adnexectomy), clear benchmarks with regard to performance scores and time per procedure, and the transfer of the acquired skills to the operating room to not only confirm the model’s great potential in surgical education but also offer a more detailed view on how to best incorporate the MaiSurge model into routine training.

### Conclusions

Surgical simulation training is of growing importance to meet the requirements of patient safety and adequate surgical skill development during residency while addressing the growing financial pressure on hospitals. The newly developed MaiSurge uterus model presents a cost-effective, realistic, and feasible training option for TLHs, thus allowing for the simulation of even advanced surgical skills outside of the operating room during gynecological residency. Furthermore, the first validity evidence for the use of the model to assess the skill level of trainees was provided in this trial.

## Supplementary material

10.2196/66369 Multimedia Appendix 1 Supplementary material, including study flowchart, modified H-OSATS description and score, and comparison of suturing time and OSATS score between novices and experts. H-OSATS: Hysterectomy–Objective Structured Assessment of Technical Skills Score; OSATS: Objective Structured Assessment of Technical Skills Score.

## Abbreviations

H-OSATS: Hysterectomy–Objective Structured Assessment of Technical Skills Score
ICC: intraclass correlation coefficient
OSATS: Objective Structured Assessment of Technical Skills Score
PVA: polyvinyl alcohol
TLH: total laparoscopic hysterectomy
