# Supplementary material for: Developing a Realistic and Cost-Effective Training Model (MaiSurge) for Laparoscopic Hysterectomies to Train and Assess Surgical Skill: Prospective Nonrandomized Controlled Trial
Source: JMIR Med Educ. 2026 Feb 12;12:e66369. doi: 10.2196/66369 (PMC12900277; doi:10.2196/66369)

**Appendix**

**Figure S1** Flowchart of the study process


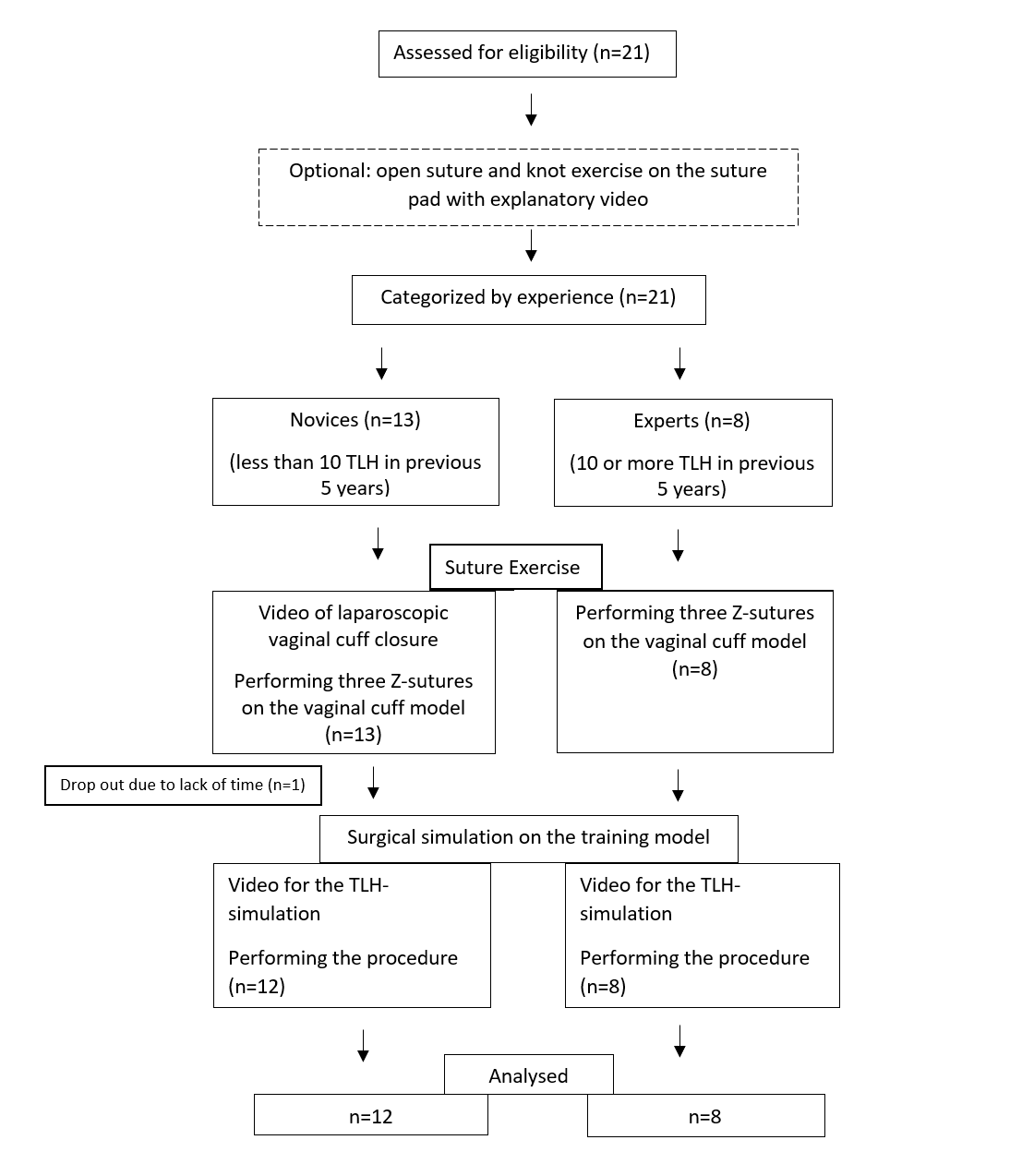


**Item S1** Modified H-OSATS score (Description)

The first assessment points of the original H-OSAT include positioning the patient, accessing the abdomen, creating a pneumoperitoneum and inspecting the abdominal organs. These points cannot be assessed for the surgical simulation on our model. Point four deals with the insertion of three trocars, where the extent to which the trocars were placed under visualisation can be assessed. In point five, the inspection of the pelvis and organs is assessed, partial points can be awarded here. In point six, the round ligaments are cut. As positioning by the manipulator in the model is only possible to a limited extent, this part cannot be assessed. The coagulation and severing of the rotundum ligament and the spreading of the two leaves of the latum ligament can be assessed. Partial points can be awarded for the transection of the ovarian ligaments (coagulation and transection) as well as considering the ureter. Point eight assesses the detachment of the peritoneum from the urinary bladder. Point nine cannot be included as there is no posterior peritoneum in the model. Partial points can be scored for the dissection of the vessels, however, the vessels in the model are not perfused, but the handling can still be scored. The separation of the uterus with the hook in point eleven can be partially assessed. For point twelve, the vaginal removal of the uterus cannot be assessed, but the closure of the vagina can be assessed. As there is no blood flow and no pneumoperitoneum, points 13 and 14 cannot be assessed. It also makes no sense to assess point 15, which contains the sequence of surgical steps, in the simulation model. This resulted in the following optimised OSATs for the simulation model (see A3). A total of 21 items were included, resulting in a maximum score of 105 points.

**Table S1** Modified H-OSATS score

| **Steps** | | | | **1** | **2** | **3** | **4** | **5** | |
| --- | --- | --- | --- | --- | --- | --- | --- | --- | --- |
| **Division of the round ligaments (left & right)** | 6.2 | Coagulation and transection of the round ligament | Performed to close to uterine horn; caused significant bleeding or tissue trauma | |  | Performed adequately, with minor bleeding |  | Performed skillfully and efficiently |  |
|  | 6.3 | Individualize the front and back fold of the anterior leaf of the broad ligament | Not performed | |  | Performed adequately, but the plan was not easily found |  | Plan developed skilfully with minimal bleeding and tissue trauma |  |
| **Division of IP ligament or utero-ovarian ligament (left & right)** | 7.2 | Expose IP ligament or utero-ovarian ligament | Inadequate exposure putting surrounding structures at risk | |  | Performed adequately |  | Performed adequately, allowing optimal exposure |  |
|  | 7.3 | Expose the posterior leaf of the broad ligament in its grey area | Unable to expose the posterior leaf | |  | Performed adequately, with minor bleeding |  | Performed skillfully and efficiently |  |
|  | 7.4 | Open a peritoneal window in the broad ligament and enlarge | Performed inadequately: did not check underlying structures or caused damage | |  | Performed adequately, in the right direction, with minor bleeding |  | Performed skillfully and efficiently, peritoneal window enlarged by divergent traction |  |
|  | 7.7 | Coagulate using an appropriate energy source or suture | Coagulated too close to surrounding structures or insufficiently | |  | Coagulated at the appropriate level, with some hesitation |  | Coagulated safely and efficiently at a 90° angle |  |
|  | 7.8 | Section the IP ligament or the utero-ovarian ligament | Section causing significant bleeding | |  | Section causing minor bleeding rapidly controlled |  | Performed skillfully at a 90° angle, causing no bleeding |  |
| **Creation of the bladder flap** | 8.2 | Open the anterior fold of the broad ligament on both sides down to the level of the vesico-uterine reflection | Performed inadequately: in the wrong plan, causing excessive bleeding, in the wrong direction | |  | Performed clumsily but in the right plan. Slow to control bleeding |  | Performed smoothly in the right plan |  |
|  | 8.4 | Section the peritoneum down to the lower uterine segment | Not performed, or caused significant bleeding or tissue trauma | |  | Performed adequately, with hesitation |  | Performed adequately in the right plan, with no bleeding |  |
|  | 8.6 | Opening of the vesico-uterine space at the midline until the cervico-vaginal margin is exposed | Opening in the wrong plan, caused significant bleeding or tissue trauma | |  | Moderate difficulty to identify correct plan, occasional bleeding |  | Opening in the correct plan down to the cervico-vaginal margin; no bleeding |  |
| **Opening of the posterior peritoneum (left & right)** | 9.2 | Dissection and section of the posterior leaf of the broad ligament towards the insertion of the utero-sacral ligaments on each side | Performed in the wrong direction or in the wrong plan. caused significant bleeding | |  | Performed adequately (Moderate difficulty to identify correct plan, occasional bleeding) |  | Performed smoothly and skillfully |  |
| **Division of the uterine vessels (left & right)** | 10.2 | Optimize exposure of the uterine vessels | Not performed | |  | Insufficient exposure |  | Excellent exposure (retracting fundus in the opposite direction) |  |
|  | 10.3 | Skeletonize uterine vessels at the ascending portion of the uterine artery | Insufficient dissection of uterine artery; caused bleeding or tissue damage | |  | Sufficient exposure of uterine artery but done with difficulty or causing moderate bleeding |  | Uterine vessels perfectly exposed, no bleeding |  |
|  | 10.5 | Coagulate the uterine vessels using an appropriate energy source or suture | Performed inadequately: at the wrong level, incorrect angle or inappropriate energy source. | |  | Performed adequately but with hesitation |  | Performed skillfully and efficiently |  |
|  | 10.6 | Section uterine vessels in the ascending portion, at the level of the colpotomizer | Section at the wrong level occasioning important bleeding or tissue trauma | |  | Performed adequately but with hesitation; occasioned bleeding but was able to control |  | Performed efficiently and skillfully, at a 90° angle |  |
|  | 10.7 | Divide cervical attachments of the cardinal ligaments | Not performed, or peformed with an incorrect angle, or causing significant bleeding or tissue trauma | |  | Performed efficiently but required some time |  | Performed skillfully and efficiently |  |
| **Colpotomy** | 11.2 | Identify the cervico-vaginal delineation from the colpotomizer | Cervico-vaginal delineation poorly identified | |  | Identified with some difficulty or incompletely |  | Cervico-vaginal delineation clearly identified |  |
|  |  |  |  |  |  |  |  |  |  |
|  | 11.3 | Check that there are no interposed elements around the vaginal fornices and complete dissection if necessary | Not performed | |  | Performed partially and hastily |  | Careful inspection, clear visualization of the distance from bladder, rectum and ureters |  |
|  | 11.4 | Proceed to circumferential colpotomy using an appropriate energy source | Performed in wrong location, not over the colpotomizer; caused excessive bleeding or tissue trauma; poor exposure | |  | Performed adequately but with difficulty, causing moderate bleeding |  | Performed skillfully over the colpotomizer on the full circumference of the vaginal fornices |  |
| **Uterus retrieval and vault closure** | 12.3 | Suture the vaginal vault with interrupted or continuous sutures | Inadequate knot tying technique, damage to surrounding structures or poor quality of suture | |  | Performed adequately, tissues generally well approximated |  | Performed skillfully and smoothly; Tissues very well approximated |  |
|  | 12.4 | Vaginal suture including sufficient width of vaginal mucosa and fascia | Suture not including both layers; Insufficient bites | |  | Performed adequately, but uneven width of vagina |  | Performed efficiently with regular good bites |  |
|  |  |  |  | |  |  |  |  |  |

**Figure S2** Comparison of suturing time between Novices and Experts


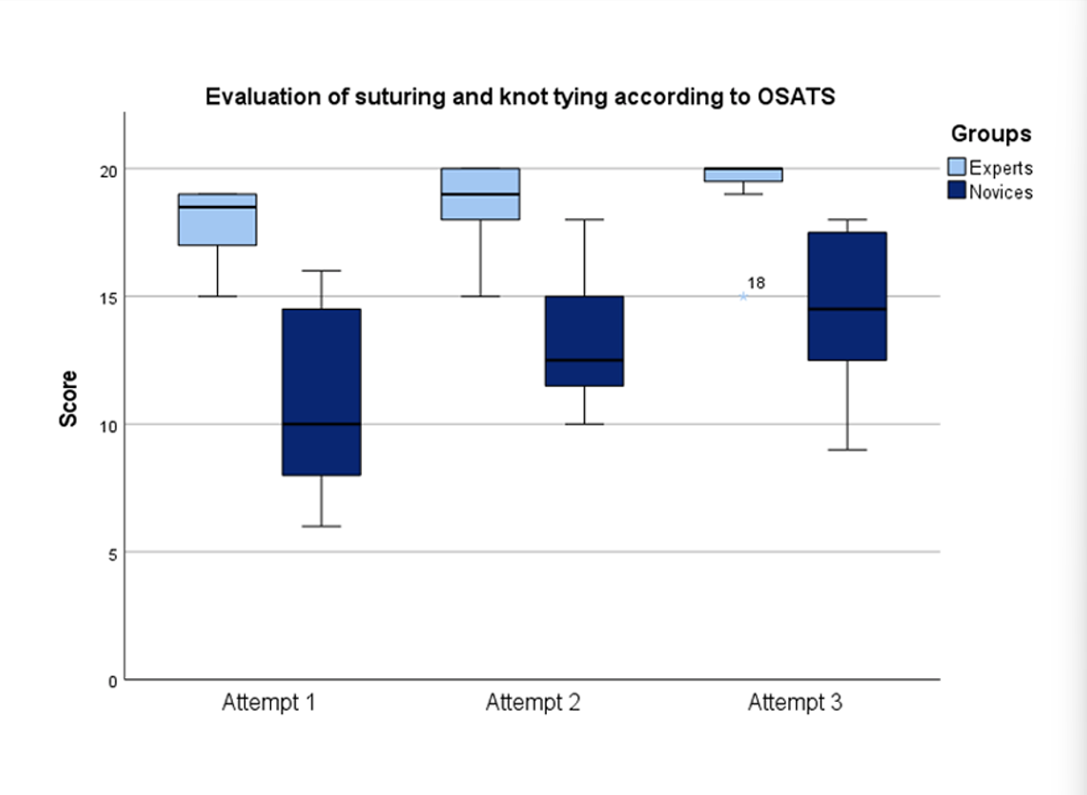


**Figure S3** Comparison of Objective Structured Assessment of Technical Skills (OSATS) score between Novices and Experts


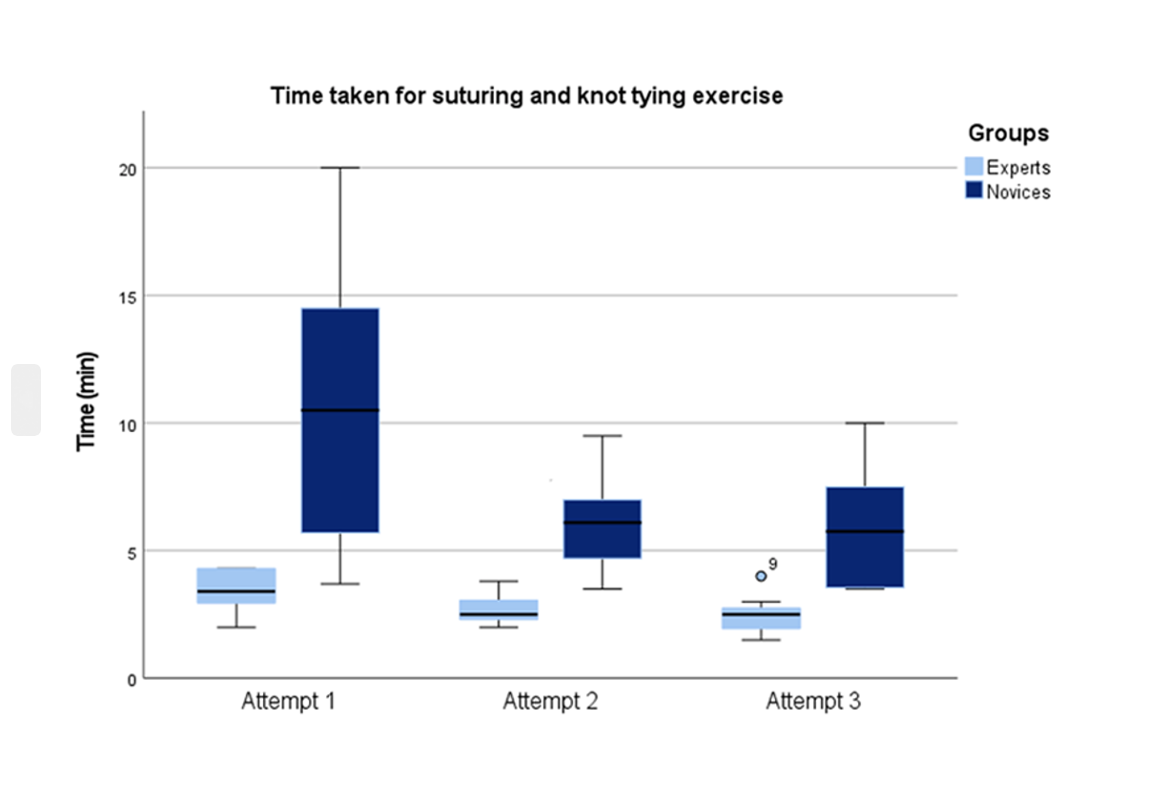

Supplement: Multimedia Appendix 1 [file mededu-v12-e66369-s001.docx]
